# Active surveillance in patients with a complete clinical response after neoadjuvant chemoradiotherapy for esophageal- and gastroesophageal junction cancer

**DOI:** 10.1515/iss-2023-0010

**Published:** 2024-11-07

**Authors:** Tamara J. Huizer, Sjoerd M. Lagarde, Joost J.M.E. Nuyttens, Lindsey Oudijk, Manon C.W. Spaander, Roelf Valkema, Bianca Mostert, Bas P.L. Wijnhoven, SANO- study group

**Affiliations:** Department of Medical Oncology, Erasmus MC Cancer Institute, Erasmus University Medical Centre, Rotterdam, The Netherlands; Department of Surgery, Erasmus MC Cancer Institute, Erasmus University Medical Centre, Rotterdam, The Netherlands; Department of Radiation Oncology, Erasmus University Medical Centre, Rotterdam, The Netherlands; Department of Pathology, Erasmus University Medical Centre, Rotterdam, The Netherlands; Department of Gastroenterology, Erasmus University Medical Centre, Rotterdam, The Netherlands; Department of Radiology and Nuclear Medicine, Erasmus University Medical Centre, Rotterdam, The Netherlands

**Keywords:** esophageal and junctional cancer, neoadjuvant chemoradiotherapy, active surveillance, gastroesophageal junction cancer, watchful waiting

## Abstract

Neoadjuvant chemoradiotherapy in patients with esophageal- and gastroesophageal junction cancer induces tumor regression. In approximately one fourth of patients, this leads to a pathological complete response in the resection specimen. Hence, active surveillance may be an alternative strategy in patients without residual disease after neoadjuvant chemoradiotherapy. Previous studies have shown that the combination of esophagogastroduodenoscopy with bite-on-bite biopsies, endoscopic ultrasound with fine needle aspiration of suspected lymph nodes, and a PET-CT-scan can be considered adequate for the detection of residual disease. So far, it has been unclear whether active surveillance with surgery as needed is a safe treatment option and leads to non-inferior overall survival compared to standard esophagectomy after neoadjuvant chemoradiotherapy. This review will discuss the current status of active surveillance for esophageal and junctional cancer.

## Introduction

The Dutch CROSS-trial showed that the addition of neoadjuvant chemoradiation (nCRT) to surgery led to an absolute 5-year overall survival (OS) benefit of 14 % without an increased risk of postoperative complications in patients with adenocarcinoma (AC) and squamous cell carcinoma (SCC) of the esophagus and gastroesophageal junction [1]. This survival benefit remained after a minimum follow-up of 10 years [2]. The Neo-AEGIS trial was initiated to compare the efficacy of nCRT (CROSS-regimen) with perioperative chemotherapy (MAGIC or FLOT regimen) in AC of the esophagus and gastroesophageal junction. Although the study was terminated early due to lack of accrual, results showed that OS after perioperative chemotherapy was similar to nCRT [3]. Based on these data, perioperative chemotherapy or nCRT are widely used in Europe in the multimodal treatment of locally advanced esophageal and junctional cancer. The ongoing ESOPEC trial (NCT02509286) investigates the direct comparison of perioperative FLOT chemotherapy and nCRT. Results are expected in 2024 [4].

After neoadjuvant chemoradiation, a higher percentage of patients have a complete pathological response. This was 16 % in the neoAEGIS trial and 29 % in the CROSS-trial compared to a 5 % pCR rate in the Neo-AEGIS trial and 16 % in the FLOT4-AIO trial [3], 5]. This relatively high rate of pCR after nCRT poses the question whether esophagectomy is needed in patients with a complete clinical response after neoadjuvant treatment. Similar to patients with a complete clinical response to chemoradiation for rectal cancer, an active surveillance strategy may be feasible. In this approach, patients undergo serial diagnostic tests after nCRT to assess residual/regrowth of cancer. Patients are offered surgery only when there is locoregional residual or recurrent disease. Active surveillance after chemoradiation for rectal cancer is a valid treatment option as 74.8 % of clinical complete responders have sustained complete clinical response after two years of follow-up [6], [7], [8]. In this review, we will focus on the concept of active surveillance in patients after nCRT for esophageal cancer as is currently being explored in the Dutch Surgery As Needed for Oesophageal cancer (SANO)-study.

## Concept of active surveillance

**Figure 1:**
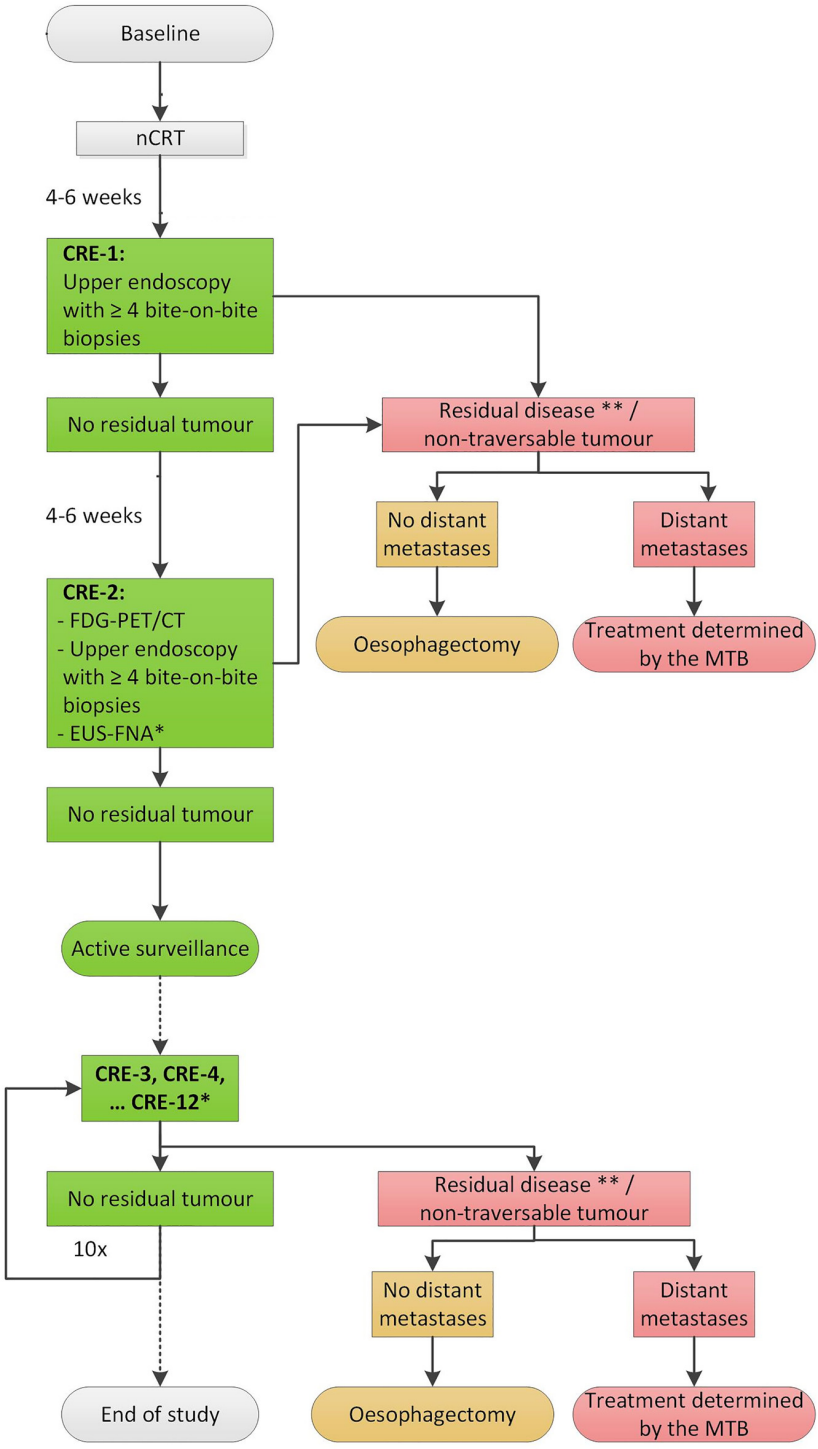
Schematic overview active surveillance strategy. CRE=Clinical Response Evaluation, EUS-FNA=Endoscopic Ultrasound with Fine Needle Aspiration, MTB=Medical Tumor Board, nCRT=Neoadjuvant Chemoradiotherapy. Amended from Ref. [51].

Active surveillance entails close observation of patients instead of standard surgery after nCRT. To identify patients that qualify for this approach, diagnostic tests are needed to assess tumor response after nCRT. The preSANO trial showed that a clinical response evaluation (CRE) using esophagogastroduodenoscopy (OGD) with bite-on-bite biopsies combined with endoscopic ultrasonography and fine-needle aspiration of suspicious lymph nodes (EUS-FNA) is adequate for the detection of locoregional residual disease after nCRT. Positron emission tomography-computed tomography (PET-CT) is used for the detection of interval metastases [9]. When there is no proven residual/recurrent disease after chemoradiation, patients will not proceed to surgery, but remain under strict surveillance. Esophagectomy is only offered to patients with residual disease or regrowth of disease after nCRT and in the absence of distant metastases. A schematic overview of active surveillance according to the SANO-study protocol is provided in Figure 1.

An organ-sparing (active surveillance) strategy may be beneficial for patients. Patients that have a persistent cCR after 5 years of follow-up are considered cured by chemoradiation alone. These patients are not exposed to the risks of esophagectomy including postoperative complications (up to 60 % of patients) and mortality (up to 3.1 %) [10], 11]. Esophagectomy is associated with a major impact on the patient’s QoL (Quality of Life). Disturbing symptoms related to the reconstruction of the foregut may last for many years and impact on patient’s wellbeing [12], 13]. Hence, preservation of the esophagus will likely lead to a better quality of life. If clinical response evaluations detect regrowth of cancer, postponed surgery is still possible. Several studies have shown that delayed surgery (>12 weeks after nCRT) is not associated with more complications or decreased survival [14].

Despite nCRT plus esophagectomy, disease recurs within 2 years in 30–40 % of patients [1], 14]. Distant metastases are seen in the majority of these patients and cure is very rare. Within an active surveillance strategy, these metastases would also develop, but patients are spared major surgery from which they will not benefit. Surgery may even hinder the possibilities for palliative anti-tumor treatment, given the high rate of postoperative morbidity.

A potential risk of the active surveillance strategy is failure of the diagnostic modalities to timely detect residual or recurrent disease. It can be hypothesized that residual disease, which remains undetected during a longer period of time, might lead to disseminated disease, and negatively affect survival. A second possible disadvantage is a lower rate of negative resection margins, due to lag time between disease recurrence and diagnostic evaluation leading to more advanced and possibly an irresectable tumor. Currently, the radical resection rate (pR0) after nCRT and esophagectomy is as high as 95 % [15].

Another potential limitation of active surveillance is the psychological impact of not opting for standard esophagectomy. Patients may experience fear of disease recurrence and anxiety due to the uncertainty if postponed esophagectomy will be necessary. On the other hand, patients are willing to reduce the chance of surviving in order to have a better quality of life without surgery [16]. Furthermore, the frequent hospital visits and invasive diagnostic tests may negatively influence the patients’ well-being [17]. These CREs may pose a clinical and economic burden on the health care system as well. It is currently not known how this economic burden translates to the costs saved by not performing esophagectomy in a subset of patients.

## Response evaluations after nCRT

Shapiro et al. demonstrated that residual tumors after nCRT are mostly seen in the mucosal, submucosal or proper muscle layer (72 , 75 and 65 %, respectively). Less frequently the surrounding stroma (42 %) or locoregional lymph nodes (37 %) are involved [18]. Furthermore, only 11 % of patients had residual tumor in the muscle layer, surrounding stroma or lymph nodes without involvement of the mucosa or submucosa. Tang and Chao et al. also demonstrated that residual disease is mainly present in the superficial layers of the esophagus after nCRT in patients with esophageal SCC. However, higher rates of residual disease in lymph nodes and/or surrounding stroma (16.6 and 17.0 %) in the absence of tumor in superficial layers were found [19], 20].

The frequent location of residual tumor in the mucosa and submucosa demonstrates the relevance of an adequate assessment of these layers during active surveillance. Endoscopy with (deep) biopsies of the tumor bed is therefore important. However, with a sensitivity of only 54 %, biopsies alone seem not accurate enough for detection of residual disease after nCRT [9], 21]. Bite-on-bite or key-hole biopsies were introduced in order to improve the accuracy of biopsies. According to this technique, a second biopsy is taken at the same location as the first biopsy. It was hypothesized that tissue biopsies could be obtained from the submucosal layer of the esopagheal wall, resulting in increased accuracy for detecting residual disease. However, van der Bogt et al. showed that increased sensitivity of bite-on-bite biopsies is most likely due to the increased number of biopsies as opposed to deeper penetration of the esophageal layers [22].

Multiple studies have investigated the diagnostic accuracy of several modalities to detect residual disease in the esophagus or gastroesophageal junction after nCRT [23]. A meta-analysis showed a summary sensitivity of OGD with classic biopsies to identify residual disease after nCRT of 33 %. The specificity, i.e. correctly classifying a patient as an incomplete responder, was 95 %. Sensitivity and specificity for PET-CT were 74 and 52 %, respectively. EUS-FNA showed the highest sensitivity (96 %), but a specificity of only 8 %. Only a few studies were well powered and prospective. Furthermore, there was substantial heterogeneity between studies. Finally, only one response assessment was performed after nCRT, whilst active surveillance entails repeated diagnostic tests (CREs) at regular time intervals.

The preSANO trial was designed to determine the accuracy of (a combination of) diagnostic modalities for the detection of residual or recurrent disease after nCRT [9]. In this prospective multicenter trial, patients with resectable SCC or AC of the esophagus underwent OGD with biopsies, and radial EUS with measurement of maximum tumor thickness and area, between 4 and 6 weeks after completion of nCRT. In case of histologically proven residual disease or no-pass during OGD, PET-CT was performed to rule out distant metastases. Patients without distant metastases underwent surgery. All patients with cCR at the first evaluation underwent a second CRE consisting of OGD with biopsies, EUS with measurement of maximum tumor thickness, PET-CT and FNA of PET-avid lesions or suspected lymph nodes at 12–14 weeks after nCRT. Subsequently, all patients without distant metastases underwent surgery. Initially, the resection specimens of nearly one third of patients classified as cCR showed >10 % residual tumor cells (Mandard tumor regression grade (TRG) 3–4). After the interim analysis for safety, the biopsy strategy during OGD was changed from conventional mucosal biopsies to bite-on-bite biopsies. With bite-on-bite biopsies and FNA, 10 % of TRG3–4 residual tumors were missed during the CRE. No adverse events related to biopsy or FNA were encountered during the trial. CRE consisting of OGD with bite-on-bite biopsies and EUS with FNA of suspicious lymph nodes was felt to be adequate for detection of >10 % residual disease (TRG3–4). PET-CT is not suitable for the evaluation of locoregional disease due to high false-positive rates, but is of value in the detection of disseminated disease. Measurement of maximum tumor thickness showed poor diagnostic accuracy, and a high frequency of false positives for TRG1 tumors (41 %) [9], 24]. The authors concluded that the combination of OGD with bite-on-bite biopsies, EUS-FNA, and PET-CT improves diagnostic accuracy of residual (locoregional and distant) disease after nCRT and could be used in clinical trials. Post-treatment endoscopic biopsy alone is a poor predictor of pathological response in patients undergoing chemoradiation therapy for esophageal cancer. The prospective, multicenter PreSINO (pre-surgery if needed for oesophageal cancer) trial was initiated in order to assess the accuracy of CREs in the detection of substantial (>10 %) residual disease in patients with SCC from Asia [25]. The CREs have been performed in a similar fashion as described in the preSANO trial. The diagnostic value of circulating tumor DNA was also assessed. The number of patients to be included was 400. Recruitment started in august 2019, and was completed by the end of 2022.

Vollenbrock et al. compared the performance of diffusion-weighted magnetic resonance imaging (DW-MRI) to PET-CT for the detection of residual disease after nCRT in patients with esophageal cancer [26]. Regardless of clinical response, PET-CT frequently shows local FDG-avidity as a result of (radiation) esophagitis. Therefore, PET-CT has a very low specificity for the assessment of local disease after nCRT. This study showed a higher diagnostic accuracy for the detection of local residual disease after nCRT with DW-MRI compared to PET-CT (sensitivity 92–96 vs. 69 %). In the future, combining these two modalities might therefore be of additional value, but these results await prospective validation [27].

## Studies on active surveillance in esophageal cancer

Seven studies,on active surveillance, including one randomized controlled trial, were included in a meta-analysis that assessed OS of patients with cCR after nCRT [28]. Patients with a cCR were offered active surveillance in case of being unfit for surgery, or upon patient request. A total of 451 patients (256 standard esophagectomy vs. 195 active surveillance) were included in the intention-to-treat population (ITT). For the per-protocol (PP) analysis, patients undergoing active surveillance or surgery were propensity score matched. Median follow-up was 63 (59–68) months for standard esophagectomy and 50 (45–56) months for active surveillance. Risk of all-cause mortality for patients undergoing active surveillance was 1.08 (ITT population, 95 % CI: 0.62–1.87, P=0.75) and 0.93 (PP population, 95 % CI: 0.56–1.54, P=0.75) compared to standard esophagectomy. No statistically significant difference in OS between patients undergoing active surveillance or standard esophagectomy was found, despite a higher proportion of medically unfit patients in the active surveillance group. On the other hand, patients that underwent active surveillance group may be referred for surgery in case of inconclusive results at CRE. Hence the active surveillance group will consist of patients with the highest probability of cCR, an independent predictor of improved survival [29]. Of importance was the finding that in 95 % of patients that underwent postponed esophagectomy for locoregional recurrence in the active surveillance group, a radical resection (R0) was achieved. This rate was similar to patients operated 4–6 weeks after completion of nCRT, suggesting no additional risk of irradical resection in case of postponed esophagectomy [15].

## Prospective studies on active surveillance

The SANO study (surgery as needed for oesophageal cancer) is a multicenter non-inferiority stepped wedge cluster randomized trial in patients with AC and SCC of the esophagus and gastroesophageal junction [30]. Non-inferiority is defined as a 3-year survival rate that is no more than 15 percentage points below the expected 3-year survival rate among patients in the standard surgery group. Randomization between nCRT plus surgery and active surveillance in patients with cCR takes place at the institutional level and not on patient level. In the active surveillance arm, patients undergo frequent CREs with OGD and bite-on-bite biopsies, EUS-FNA and PET-CT; every three months in year 1, every four months in year 2, every six months in year 3, and once per year in year 4 and 5 of follow-up. Patients without residual disease at the end of follow-up are considered cured. Inclusion of patients in the SANO trial was completed in December 2020, and the first results on the primary endpoint of two-year overall survival are expected late 2023. The DSMB monitored multiple parameters in order to ensure the safety of patients. These include the proportion of patients in the active surveillance arm who develop irresectable/incurable recurrence, the proportion of irradical (R1) resections, and the proportion of distant metastases in both treatment arms. So far no stopping rules have been violated.

During the trial we observed that one fourth of patients in the standard (immediate surgery) arm refused esophagectomy, and switched to active surveillance. Apparently, patients supported the idea of active surveillance when there are no signs of residual disease at 12 weeks after nCRT. Therefore it was felt that after closure of the SANO trial, patients should still be offered active surveillance as an alternative to standard surgery. The SANO-2 study, a multicenter, prospective observation extension study on active surveillance was initiated in March 2021. The goal of this trial is to monitor the safety, implementation and efficacy of active surveillance outside the SANO trial. Furthermore, all patients eligible for active surveillance in the SANO-2 cohort are offered decision counselling, and receive questionnaires on quality of life and regret of their decision, to undergo either standard esophagectomy or active surveillance. The SANO-2 will continue until the SANO-trial data are available.

Another randomized controlled trial on active surveillance, is the phase III multicenter ESOSTRATE-trial (comparison of systematic surgery vs. surveillance and rescue surgery in operable esophageal cancer with a complete clinical response to radiochemotherapy), and is currently recruiting [31]. The primary outcome is disease free survival (DFS) at 2 years, hypothesizing superiority of direct surgery vs. active surveillance (2-year DFS rate: 45 vs. 30 %). A response evaluation is performed at 5–6 weeks after nCRT. Patients with cCR are randomized to either direct surgery or active surveillance. Since the protocol of the ESOSTRATE study is currently not publically available, the details on diagnostic modalities used for response evaluation, and the frequency in which they are being performed, is unknown. Of note, the ESOSTRATE is a superiority trial and not designed to show equivalence of non-inferiority of active surveillance. There is a risk of imbalance for confounders between the two study arms in a cluster randomized trial (SANO-trial) and more patients need to be included to achieve similar statistical power compared to individual randomization. In cluster randomization patients are aware of the assigned treatment at the time of informed consent and this may lead to a better accrual when comparing a surgical and non-surgical treatment. Furthermore, cluster randomization might be more efficient, since only one treatment is offered (active surveillance or direct surgery) at any particular time [32].

PD-1 (programmed cell death protein 1) is expressed on T-cells and act as immunomodulator. Binding of its ligand PD-L1 (expressed on a subset of immune cells, antigen-presenting cells and tumor cells) to PD-1 induces apoptosis of antigen-specific T-cells, and suppresses apoptosis of regulatory T-cells. Blockage of the PD1 axis by an anti-PD-1/PD-L1/PD-L2 antibody prevents the inhibition of the immune response, leading to enhanced tumor cell killing by increased T-cell activation and proliferation [33]. In the adjuvant and in the metastatic disease setting, treatment with immunotherapy monotherapy or in addition to chemotherapy, results in improved DFS and OS [34], [35], [36], [37]. Recent small studies in esophageal cancer have demonstrated that CRT combined with immunotherapy can significantly improve tumor regression and the rate of cCR, which might increase the number of patients eligible for active surveillance. This approach is currently being investigated in the WATCHER trial (watch and wait for neoadjuvant concurrent radiochemotherapy combined with camrelizumab in patients with resectable ESCC [38]). In this prospective randomized controlled phase II trial, nCRT is combined with the anti-PD-1 monoclonal antibody camrelizumab. In total, 100 patients with locally advanced esophageal SCC receive neoadjuvant immunochemoradiotherapy (iCRT). Six weeks after completion of the neoadjuvant treatment, all patients undergo CRE. Patients with cCR are randomized to direct surgery or active surveillance. Patients in the active surveillance arm undergo a second evaluation (CRE2), and are frequently evaluated (CRE3-14) in case of persistent complete response. All patients who were considered complete responder at some time point, receive 14 cycles of camrelizumab q3w maintenance therapy, either after radical surgery or during active surveillance. No detailed information on the clinical assessment of response is available, since the study protocol has not been published. The primary outcome measure is 1-year DFS in patients with a cCR at CRE1 after neoadjuvant iCRT. Recruitment started in August 2022, and study completion is expected in 2027.

### Future perspectives

Unfortunately, the vast majority of patients after nCRT are not eligible for an active surveillance approach, since cCR is achieved in approximately 30 % of patients. The addition of immunotherapy to nCRT, simultaneously or sequentially, has the potential to increase the proportion of patients with a cCR, and thereby increase the proportion of patients eligible for active surveillance.

There are several phase II, mostly single arm studies ongoing that combine nCRT with perioperative immunotherapy [39]. A single center phase I/II trial evaluates the safety, tolerability and efficacy of avelumab (anti PD-L1 monocloncal antibody) in combination with nCRT [40]. Furthermore, immunotherapy can be administered during nCRT as extended neoadjuvant treatment. The Phase Ib PALACE-1 trial showed that preoperative pembrolizumab combined with nCRT in patients with ESCC was safe [41]. Furthermore, a pCR rate of 55.6 % was achieved in this small cohort of 20 patients. In the phase II PALACE-2 trial, all patients are treated with concomitant nCRT and pembrolizumab. Esophagectomy will take place 4–6 weeks after completion of nCRT [42]. The phase II single arm PROCEED trial also investigates the addition of pembrolizumab to nCRT, albeit in a slightly different dosing interval (pembrolizumab 2 weeks prior, within the first, and at the fourth week of nCRT) [43]. Primary outcome in both trials is pCR.

Another opportunity to reduce the number of patients needing esophagectomy, is the addition of immunotherapy (nivolumab) during active surveillance. Approximately two-thirds of patients with cCR undergoing active surveillance, develop recurrent disease (preliminary results SANO trial). This demonstrates the need for effective maintenance therapy in order to reduce the number of patients with recurrent disease. Previous work demonstrated an increased DFS in patients who were treated with adjuvant nivolumab after nCRT and esophagectomy [34]. The phase IIb SANO-3 study aims to evaluate DFS in patients with cCR at CREs in the SANO-2 study [44]. Patients receive nivolumab q4w for a maximum of 1 year, or until disease recurrence. Recruitment started in 2022, and results are expected in 2026.

Several promising methods to improve the accuracy of detection of residual- or recurrent disease have been proposed. A technique which might prove to be of additional value in assessing tumor response is the analysis of circulating tumor DNA (ctDNA). This type of cell-free DNA can be found in the blood circulation (and other biofluids such as urine and saliva), and is released by the tumor cells after necrosis or apoptosis [45]. Changes in ctDNA quantity can be used for non-invasive evaluation of treatment response and detection of recurrence. This is an attractive diagnostic test within active surveillance which currently calls for repeated invasive diagnostic procedures. A recent analysis showed that the presence of ctDNA in esophageal cancer patients was associated with the development of metastases and decreased disease-specific survival [45], 46]. A recent meta-analysis of multiple prospective studies demonstrated that ctDNA is a suitable biomarker for both diagnosing and monitoring esophageal cancer, predominantly in metastatic disease [47]. Less is known about the value of ctDNA in detecting residual or recurrent disease after neoadjuvant treatment and during active surveillance [48]. However, there are technical challenges hindering the use of ctDNA in current practice, mainly due to the low concentration of ctDNA in blood. Future studies will demonstrate if these modalities can further improve the ability to detect residual or recurrent disease at CREs.

Lastly, there is room for improvement within the currently used diagnostic modalities for the detection of residual or recurrent disease. Posthoc analysis of preSANO data showed EUS at 10–12 weeks after nCRT detected only half of the patients with residual nodal disease [24]. It is suggested the EUS criteria to define suspicious lymph nodes may be less applicable after nCRT due to postradial fibrosis and inflammation [49]. These criteria might be adapted in the future to improve diagnostic accuracy of EUS after nCRT.

In conclusion, several prospective studies will soon demonstrate if active surveillance is a safe and effective treatment strategy for patients with cCR after nCRT [50]. Intensifying multimodality treatment (e.g. aiming for more clinical complete responders), and improved detection of micrometastases may reduce the role and need of surgical intervention.

## References

[1] Shapiro J, Van Lanschot JJB, Hulshof M, Van Hagen P, Van Berge Henegouwen MI, Wijnhoven BPL (2015). Neoadjuvant chemoradiotherapy plus surgery versus surgery alone for oesophageal or junctional cancer (CROSS): long-term results of a randomised controlled trial. Lancet Oncol.

[2] Eyck BM, Van Lanschot JJB, Hulshof M, Van der Wilk BJ, Shapiro J, Van Hagen P (2021). Ten-year outcome of neoadjuvant chemoradiotherapy plus surgery for esophageal cancer: the randomized controlled CROSS trial. J Clin Oncol.

[3] John VR, Shaun RP, Brian ON, Maeve Aine L, Lene B, Thomas C (2021). Neo-AEGIS (neoadjuvant trial in adenocarcinoma of the esophagus and esophago-gastric junction international study): preliminary results of phase III RCT of CROSS versus perioperative chemotherapy (modified MAGIC or FLOT protocol). (NCT01726452). J Clin Oncol.

[4] Hoeppner J, Lordick F, Brunner T, Glatz T, Bronsert P, Rothling N (2016). ESOPEC: prospective randomized controlled multicenter phase III trial comparing perioperative chemotherapy (FLOT protocol) to neoadjuvant chemoradiation (CROSS protocol) in patients with adenocarcinoma of the esophagus (NCT02509286). BMC Cancer.

[5] Al-Batran SE, Hofheinz RD, Pauligk C, Kopp HG, Haag GM, Luley KB (2016). Histopathological regression after neoadjuvant docetaxel, oxaliplatin, fluorouracil, and leucovorin versus epirubicin, cisplatin, and fluorouracil or capecitabine in patients with resectable gastric or gastro-oesophageal junction adenocarcinoma (FLOT4-AIO): results from the phase 2 part of a multicentre, open-label, randomised phase 2/3 trial. Lancet Oncol.

[6] Lopez-Campos F, Martin-Martin M, Fornell-Perez R, Garcia-Perez JC, Die-Trill J, Fuentes-Mateos R (2020). Watch and wait approach in rectal cancer: current controversies and future directions. World J Gastroenterol.

[7] Wang QX, Zhang R, Xiao WW, Zhang S, Wei MB, Li YH (2021). The watch-and-wait strategy versus surgical resection for rectal cancer patients with a clinical complete response after neoadjuvant chemoradiotherapy. Radiat Oncol.

[8] Van der Valk MJM, Hilling DE, Bastiaannet E, Meershoek-Klein Kranenbarg E, Beets GL, Figueiredo NL (2018). Long-term outcomes of clinical complete responders after neoadjuvant treatment for rectal cancer in the International Watch & Wait Database (IWWD): an international multicentre registry study. Lancet.

[9] Noordman BJ, Spaander MCW, Valkema R, Wijnhoven BPL, Van Berge Henegouwen MI, Shapiro J (2018). Detection of residual disease after neoadjuvant chemoradiotherapy for oesophageal cancer (preSANO): a prospective multicentre, diagnostic cohort study. Lancet Oncol.

[10] Pu S, Chen H, Zhou C, Yu S, Liao X, Zhu L (2021). Major postoperative complications in esophageal cancer after minimally invasive esophagectomy compared with open esophagectomy: an updated meta-analysis. J Surg Res.

[11] Kosinski AS, Raymond DP, Magee MJ, DeCamp MM, Farjah F, Society of Thoracic Surgeons General Thoracic Surgery Database Task F (2017). The society of thoracic surgeons composite score for evaluating esophagectomy for esophageal cancer. Ann Thorac Surg.

[12] Markar SR, Zaninotto G, Castoro C, Johar A, Lagergren P, Elliott JA (2022). Lasting symptoms after esophageal resection (LASER): European multicenter cross-sectional study. Ann Surg.

[13] Van der Wilk BJ, Spronk I, Noordman BJ, Eyck BM, Haagsma JA, Coene PLO (2022). Preferences for active surveillance or standard oesophagectomy: discrete-choice experiment. Br J Surg.

[14] Shapiro J, Van Hagen P, Lingsma HF, Wijnhoven BP, Biermann K, Ten Kate FJ (2014). Prolonged time to surgery after neoadjuvant chemoradiotherapy increases histopathological response without affecting survival in patients with esophageal or junctional cancer. Ann Surg.

[15] Van Hagen P, Hulshof MC, Van Lanschot JJ, Steyerberg EW, Van Berge Henegouwen MI, Wijnhoven BP (2012). Preoperative chemoradiotherapy for esophageal or junctional cancer. N Engl J Med.

[16] Noordman BJ, De Bekker-Grob EW, Coene P, Van der Harst E, Lagarde SM, Shapiro J (2018). Patients’ preferences for treatment after neoadjuvant chemoradiotherapy for oesophageal cancer. Br J Surg.

[17] Hermus M, Van der Wilk BJ, Chang RTH, Collee G, Noordman BJ, Coene PLO (2023). Patient preferences for active surveillance vs standard surgery after neoadjuvant chemoradiotherapy in oesophageal cancer treatment: the NOSANO-study. Int J Cancer.

[18] Shapiro J, Ten Kate FJ, Van Hagen P, Biermann K, Wijnhoven BP, Van Lanschot JJ (2013). Residual esophageal cancer after neoadjuvant chemoradiotherapy frequently involves the mucosa and submucosa. Ann Surg.

[19] Tang H, Jiang D, Zhang S, Zeng Z, Tan L, Hou Y (2021). Residual tumor characteristics of esophageal squamous cell carcinoma after neoadjuvant chemoradiotherapy. J Thorac Cardiovasc Surg.

[20] Chao YK, Chang Y, Yeh CJ, Chang HK, Tseng CK, Chuang WY (2016). Characterization of residual tumours at the primary site in patients with a near pathological complete response after neoadjuvant chemoradiotherapy for oesophageal cancer. Br J Surg.

[21] Van der Bogt RD, Van der Wilk BJ, Nikkessen S, Krishnadath KK, Schoon EJ, Oostenbrug LE (2021). Predictive value of endoscopic esophageal findings for residual esophageal cancer after neoadjuvant chemoradiotherapy. Endoscopy.

[22] Van der Bogt RD, Van der Wilk BJ, Oudijk L, Schoon EJ, Van Lijnschoten G, Corporaal S (2022). Bite-on-bite biopsies for the detection of residual esophageal cancer after neoadjuvant chemoradiotherapy. Endoscopy.

[23] Eyck BM, Onstenk BD, Noordman BJ, Nieboer D, Spaander MCW, Valkema R (2020). Accuracy of detecting residual disease after neoadjuvant chemoradiotherapy for esophageal cancer: a systematic review and meta-analysis. Ann Surg.

[24] Van der Bogt RD, Noordman BJ, Krishnadath KK, Roumans CAM, Schoon EJ, Oostenbrug LE (2019). Endoscopic ultrasound measurements for detection of residual disease after neoadjuvant chemoradiotherapy for esophageal cancer. Endoscopy.

[25] Zhang X, Eyck BM, Yang Y, Liu J, Chao YK, Hou MM (2020). Accuracy of detecting residual disease after neoadjuvant chemoradiotherapy for esophageal squamous cell carcinoma (preSINO trial): a prospective multicenter diagnostic cohort study. BMC Cancer.

[26] Vollenbrock SE, Voncken FEM, Lambregts DMJ, Maas M, Donswijk ML, Vegt E (2021). Clinical response assessment on DW-MRI compared with FDG-PET/CT after neoadjuvant chemoradiotherapy in patients with oesophageal cancer. Eur J Nucl Med Mol Imag.

[27] Borggreve AS, Goense L, Van Rossum PSN, Heethuis SE, Van Hillegersberg R, Lagendijk JJW (2020). Preoperative prediction of pathologic response to neoadjuvant chemoradiotherapy in patients with esophageal cancer using ^18^F-FDG PET/CT and DW-MRI: a prospective multicenter study. Int J Radiat Oncol Biol Phys.

[28] Van der Wilk BJ, Eyck BM, Hofstetter WL, Ajani JA, Piessen G, Castoro C (2022). Chemoradiotherapy followed by active surveillance versus standard esophagectomy for esophageal cancer: a systematic review and individual patient data meta-analysis. Ann Surg.

[29] Alnaji RM, Du W, Gabriel E, Singla S, Attwood K, Nava H (2016). Pathologic complete response is an independent predictor of improved survival following neoadjuvant chemoradiation for esophageal adenocarcinoma. J Gastrointest Surg.

[30] Eyck BM, Van der Wilk BJ, Noordman BJ, Wijnhoven BPL, Lagarde SM, Hartgrink HH (2021). Updated protocol of the SANO trial: a stepped-wedge cluster randomised trial comparing surgery with active surveillance after neoadjuvant chemoradiotherapy for oesophageal cancer. Trials.

[31] ClinicalTrials.gov Internet. Bethesda (MD): National Library of Medicine (US) (2000). Identifier NCT02551458, comparison of systematic surgery versus surveillance and Rescue surgery in operable oesophageal cancer with a complete clinical response to Radiochemotherapy (Esostrate); 2015 September 16.

[32] World Health Organization (2021). WHO guidance on research methods for health emergency and disaster risk management.

[33] Waldman AD, Fritz JM, Lenardo MJ (2020). A guide to cancer immunotherapy: from T cell basic science to clinical practice. Nat Rev Immunol.

[34] Kelly RJ, Ajani JA, Kuzdzal J, Zander T, Van Cutsem E, Piessen G (2021). Adjuvant Nivolumab in resected esophageal or gastroesophageal junction cancer. N Engl J Med.

[35] Sun JM, Shen L, Shah MA, Enzinger P, Adenis A, Doi T (2021). Pembrolizumab plus chemotherapy versus chemotherapy alone for first-line treatment of advanced oesophageal cancer (KEYNOTE-590): a randomised, placebo-controlled, phase 3 study. Lancet.

[36] Doki Y, Ajani JA, Kato K, Xu J, Wyrwicz L, Motoyama S (2022). Nivolumab combination therapy in advanced esophageal squamous-cell carcinoma. N Engl J Med.

[37] Janjigian YY, Shitara K, Moehler M, Garrido M, Salman P, Shen L (2021). First-line nivolumab plus chemotherapy versus chemotherapy alone for advanced gastric, gastro-oesophageal junction, and oesophageal adenocarcinoma (CheckMate 649): a randomised, open-label, phase 3 trial. Lancet.

[38] ClinicalTrials.gov Internet. Bethesda (MD): National Library of Medicine (US) (2000). Identifier NCTNCT05507411, watch and wait for NeoAdjuvant concurrent Radiochemotherapy combined with camrelizumab in patients with resectable ESCC (WATCHER); 19 August 2022.

[39] Yan Y, Feng X, Li C, Lerut T, Li H (2022). Treatments for resectable esophageal cancer: from traditional systemic therapy to immunotherapy. Chin Med J (Engl.).

[40] ClinicalTrials.gov Internet. Bethesda (MD): National Library of Medicine (US) (2000). Identifier NCT03490292, avelumab with chemoradiation for stage II/III resectable esophageal and gastroesophageal cancer; 2018 April 16.

[41] Li C, Zhao S, Zheng Y, Han Y, Chen X, Cheng Z (2021). Preoperative pembrolizumab combined with chemoradiotherapy for oesophageal squamous cell carcinoma (PALACE-1). Eur J Cancer.

[42] ClinicalTrials.gov Internet. Bethesda (MD): National Library of Medicine (US) (2000). Identifier NCT04435197, pre-operative pembrolizumab + chemoradiation in patients with locally advanced esophageal squamous cell carcinoma (PALACE-2); 2022 June 17.

[43] ClinicalTrials.gov Internet. Bethesda (MD): National Library of Medicine (US) (2000). Identifier NCT03064490 pembrolizumab, radiotherapy, and chemotherapy in neoadjuvant treatment of malignant Esophago-gastric diseases (PROCEED); 2017 February 27.

[44] ClinicalTrials.gov Internet. Bethesda (MD): National Library of Medicine (US) (2000). Identifier NCT05491616 nivolumab during active surveillance after neoadjuvant chemoradiation for esophageal cancer: SANO-3 study (SANO-3). 2022 August 8.

[45] Min J, Zhou H, Jiang S, Yu H (2022). A review of circulating tumor DNA in the diagnosis and monitoring of esophageal cancer. Med Sci Monit.

[46] Azad TD, Chaudhuri AA, Fang P, Qiao Y, Esfahani MS, Chabon JJ (2020). Circulating tumor dna analysis for detection of minimal residual disease after chemoradiotherapy for localized esophageal cancer. Gastroenterology.

[47] Chidambaram S, Markar SR (2022). Clinical utility and applicability of circulating tumor DNA testing in esophageal cancer: a systematic review and meta-analysis. Dis Esophagus.

[48] Eyck BM, Jansen MP, Noordman BJ, Atmodimedjo PN, Van der Wilk BJ, Martens JW (2023). Detection of circulating tumour DNA after neoadjuvant chemoradiotherapy in patients with locally advanced oesophageal cancer. J Pathol.

[49] Zuccaro G, Rice TW, Goldblum J, Medendorp SV, Becker M, Pimentel R (1999). Endoscopic ultrasound cannot determine suitability for esophagectomy after aggressive chemoradiotherapy for esophageal cancer. Am J Gastroenterol.

[50] De Bekker-Grob EW, Niers EJ, Van Lanschot JJ, Steyerberg EW, Wijnhoven BP (2015). Patients’ preferences for surgical management of esophageal cancer: a discrete choice experiment. World J Surg.

[51] Van der Zijden CJ, Lagarde SM, Hermus M, Kranenburg LW, Van Lanschot JJB, Mostert B (2023). A prospective cohort study on active surveillance after neoadjuvant chemoradiotherapy for esophageal cancer: protocol of surgery as needed for oesophageal cancer-2. BMC Cancer.

